# Metagenomic analysis of viruses associated with maize lethal necrosis in Kenya

**DOI:** 10.1186/s12985-018-0999-2

**Published:** 2018-05-23

**Authors:** Mwathi Jane Wamaitha, Deepti Nigam, Solomon Maina, Francesca Stomeo, Anne Wangai, Joyce Njoki Njuguna, Timothy A. Holton, Bramwel W. Wanjala, Mark Wamalwa, Tanui Lucas, Appolinaire Djikeng, Hernan Garcia-Ruiz

**Affiliations:** 1 Kenya Agricultural and Livestock Research Organization (KALRO), P. O. Box 14733-00800, Nairobi, Kenya; 2 Department of Plant Pathology and Nebraska Center for Virology, University of Nebraska- Lincoln, Lincoln, NE 68583 USA; 3 School of Agriculture and Environment and UWA Institute of Agriculture, Faculty of Science, The University of Western Australia, 35 Stirling Highway, Crawley, WA 6009 Australia; 4 Cooperative Research Centre for Plant Biosecurity, Canberra, ACT 2617 Australia; 5 Biosciences Eastern and Central Africa-International Livestock Research Institute (BecA-ILRI), Hub, Nairobi, Kenya; 6 Plant Innovation Centre, Post-Entry Quarantine, Department of Agriculture and Water Resources, 135 Donnybrook Road, Mickleham, VIC 3064 Australia; 7 Centre for Tropical Livestock Genetics and Health (CTLGH), The University of Edinburgh, Edinburgh, Scotland EH25 9RG UK

**Keywords:** Maize lethal necrosis, MCMV, SCMV, MYDV-RMV, MSV, Metagenomics, Phylogenetics, Coat protein variation

## Abstract

**Background:**

Maize lethal necrosis is caused by a synergistic co-infection of *Maize chlorotic mottle virus* (MCMV) and a specific member of the *Potyviridae*, such as *Sugarcane mosaic virus* (SCMV), *Wheat streak mosaic virus* (WSMV) or *Johnson grass mosaic virus* (JGMV). Typical maize lethal necrosis symptoms include severe yellowing and leaf drying from the edges. In Kenya, we detected plants showing typical and atypical symptoms. Both groups of plants often tested negative for SCMV by ELISA.

**Methods:**

We used next-generation sequencing to identify viruses associated to maize lethal necrosis in Kenya through a metagenomics analysis. Symptomatic and asymptomatic leaf samples were collected from maize and sorghum representing sixteen counties.

**Results:**

Complete and partial genomes were assembled for MCMV, SCMV, *Maize streak virus* (MSV) and *Maize yellow dwarf virus*-RMV (MYDV-RMV). These four viruses (MCMV, SCMV, MSV and MYDV-RMV) were found together in 30 of 68 samples. A geographic analysis showed that these viruses are widely distributed in Kenya. Phylogenetic analyses of nucleotide sequences showed that MCMV, MYDV-RMV and MSV are similar to isolates from East Africa and other parts of the world. Single nucleotide polymorphism, nucleotide and polyprotein sequence alignments identified three genetically distinct groups of SCMV in Kenya. Variation mapped to sequences at the border of NIb and the coat protein. Partial genome sequences were obtained for other four potyviruses and one polerovirus.

**Conclusion:**

Our results uncover the complexity of the maize lethal necrosis epidemic in Kenya. MCMV, SCMV, MSV and MYDV-RMV are widely distributed and infect both maize and sorghum. SCMV population in Kenya is diverse and consists of numerous strains that are genetically different to isolates from other parts of the world. Several potyviruses, and possibly poleroviruses, are also involved.

**Electronic supplementary material:**

The online version of this article (10.1186/s12985-018-0999-2) contains supplementary material, which is available to authorized users.

## Background

Maize (*Zea mays* L.) is one of the most important cereals in Sub-Saharan Africa and is grown in approximately 25 million hectares [1]. Maize is consumed as a preferred calorie source by 95% of the population, at an average of 1075 kcal/capita/day, which represents more than 50% of the recommended daily intake [2]. Maize production is destined for human consumption or animal feed at a proportion of 88 and 12%, respectively [3, 4].

In 2011 maize lethal necrosis disease was first detected in Kenya [5–7], and confirmed in several countries in East and Central Africa, specifically in Tanzania, Uganda [8], Rwanda [9] DR Congo [10], Ethiopia and South Sudan [11]. Corn lethal necrosis (CLN) was first described in the State of Kansas in 1978 [12]. In their original descriptions, corn lethal necrosis and maize lethal necrosis defined the same disease. Herein we use maize lethal necrosis disease.

In Sub-Saharan Africa, smallholder farms account for approximately 80% of the farm land and employ 175 million people directly [13, 14]. Small-scale farmers largely rely on maize, as a major source of energy and revenue [15]. With yield losses ranging from 30 to 100% that lead to food shortages and contribute to hunger and malnutrition [16], maize lethal necrosis is currently a threat to maize production and food security in Sub-Saharan Africa.

Maize lethal necrosis is caused by a synergistic co-infection of MCMV, a Machlomovirus in the family *Tombusviridae* [17], and specific members of the family *Potyviridae*, such as SCMV [12], *Wheat streak mosaic virus* (WSMV) [18], or JGMV [19]. In maize lethal necrosis outbreaks, MCMV and SCMV is the most prevalent virus combination [9, 10, 20]. In Rwanda, *Maize yellow mosaic virus* (MaYMV), a polerovirus, was recently detected in maize plants showing symptoms similar to those caused by maize lethal necrosis [21].

Typical maize lethal necrosis symptoms include severe yellowing and leaf drying from the edges, stunting and premature plant death, sterility in male plants, poor tasseling, lack of or only a few grains in the cob, malformed or rotten cobs [7, 19]. In farmer’s fields in Kenya, we detected plants showing bright yellow stripes with green edges, which deviate from typical maize lethal necrosis symptoms. Additionally, symptomatic plants often tested negative for SCMV by ELISA, as described by others [19, 21, 22].

Maize lethal necrosis continues to spread rampantly and is a major concern to maize stakeholders [5] including small and large-scale farmers, commercial seed sector, millers, transporters, policy makers, local and international communities. These raises several questions such as why is maize lethal necrosis still difficult to manage and what strategies can farmers implement?

Natural and engineered genetic resistance provide a successful approach to managing viral diseases [23]. With respect to natural genetic resistance, massive screens of commercial hybrids and thousands of maize lines reported high levels of susceptibility. Only few lines were moderately resistant [24, 25]. Several efforts are underway to identify and characterize maize resistance to MCMV [26] and SCMV [27].

We hypothesized that uncharacterized viruses synergistically interact with MCMV to cause maize lethal necrosis, and there is genetic variation between SCMV and MCMV in East Africa compared to the rest of the world. To test these hypotheses, we collected samples from symptomatic and asymptomatic maize leaves in sixteen counties in Kenya. Cultivated and wild sorghum [*Sorghum bicolor* (L.) Moench] and napier grass (*Pennisetum purpureum* S.) were also included to determine their potential as alternate hosts. Viruses present were identified by metagenomics using next-generation sequencing of total RNA and bioinformatics. Viral presence was determined for each individual sample using *de-novo* assembled contigs.

After *de-novo* assembly, complete and partial genomes were obtained for MCMV, SCMV, *Maize yellow dwarf virus*-RMV (MYDV-RMV) and *Maize streak virus* (MSV). Partial genomes were assembled for other four potyviruses and one polerovirus. A geographic analysis showed the wide distribution of MCMV, SCMV, MYDV-RMV and MSV infecting maize and sorghum in Kenya. A large number (30/68) of the samples analyzed had a combination of four viruses: MCMV, SCMV, MYDV-RMV, and MSV. Only one sample had MCMV in the absence of other viruses. All the other samples (67/68) had MCMV plus one, two, three, or four other viruses. Phylogenetic analyses of near complete genome nucleotide sequences showed that MCMV, MSV and MYDV-RMV in Kenya are similar to isolates from East Africa. In contrast, SCMV from Kenya exhibits the largest genetic variation and distance with respect to isolates from others parts of the world, including East Africa. These results provide a solid foundation to develop virus diagnostic protocols, management strategies, and raise the possibility of a synergistic interaction between MCMV and a polerovirus to cause maize lethal necrosis.

## Methods

### Sample collection

**Fig. 1:**
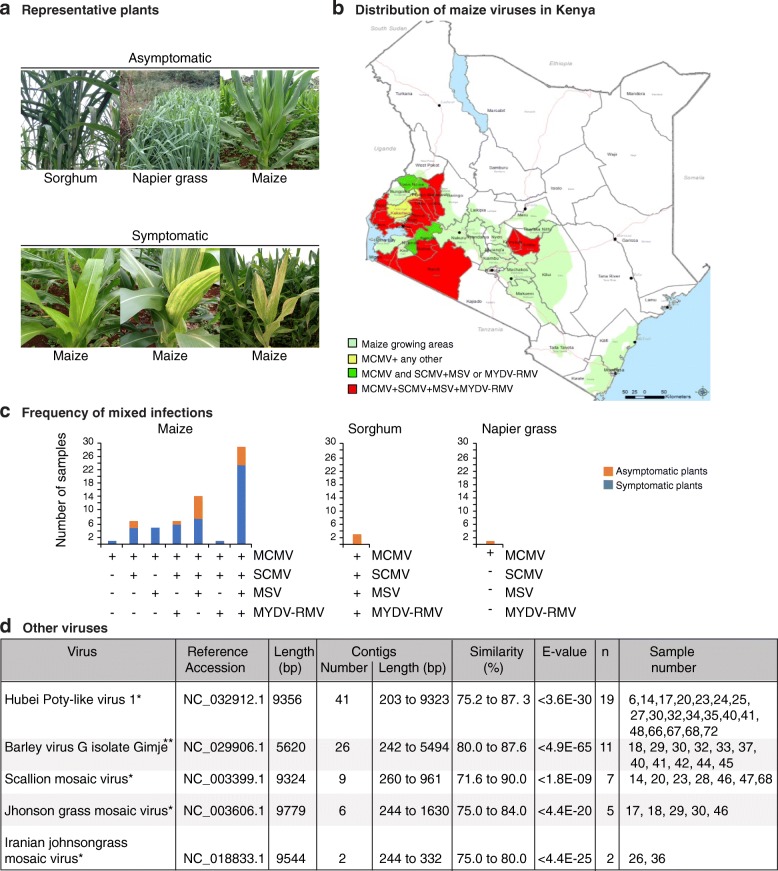
Geographic distribution of maize-infecting viruses in Kenya. **a** Representative pictures of asymptomatic and symptomatic plants sampled in this study. **b** Maize-growing areas and distribution of the main maize viruses detected in this study. Counties are color-coded to illustrate the combinations of viruses found. **c** Most abundant viruses detected and frequency of mixed infections in asymptomatic and symptomatic plants (68 samples total). **d** Other viruses detected in this study. Potyvirus and polerovirus are denoted by * and **, respectively. Reference accession number and length are provided. Number of *de-novo* assembled contigs, range of length and similarity to the reference genome is provided. Identity of samples contributing at least one contig is indicated

Between 2012 and 2014 leaf samples (0.5 g) of maize, sorghum or napier grass were collected at vegetative stage from farmer’s fields in sixteen counties in Kenya (Fig. 1). At the time of tissue collection, some plants were asymptomatic and others were symptomatic (Fig. 1a). The symptomatic plants ranged from yellow spotting (early-stage), streaking (mid-stage) or necrosis of the leaf margin (late-stage). In some cases, both, asymptomatic (20) and symptomatic (48) samples were collected from the same farm or nearby. Counties included in the sampling were selected based on yield losses caused by maize lethal necrosis (30–100%) [7, 11, 20], and were classified as maize lethal necrosis hotspots (Bomet, Narok, Nandi, Nyamira and Busia), moderate-severe hotspots (Homabay, Transzoia, Migori, Siaya, Uasin Gishu, Kisumu, Elgeyo Marakwet and Kericho) and low-medium hotspots (Embu, Kakamega and Kirinyaga). Samples were frozen in liquid nitrogen and transported to Kenya Agricultural and Livestock Research Organization (KALRO) Kabete, and stored at − 80 °C until processed.

### Geographic distribution

Geographic coordinates (latitude and longitude) of the 68 sample locations were marked by Global Positioning System (GPS) and linked to viruses found. Data was converted into GIS using ARCGIS 10.4. Geographical distribution of the samples collected and the viruses found (68 data points for 68 samples) was constructed by overlaying the layer of sample points with that of the maize growing zones within Kenya forming the background layer. Counties sampled were color-coded based on the combinations of viruses found (Fig. 1b). The samples were identified by county of origin a consecutive number (3 through 72).

### Total RNA extraction

RNA was extracted from 0.1 g of leaf tissue using ZR Plant RNA MiniPrep™ (Catalog No. R2024) according to the manufactures instructions. In brief, tissue was ground with a pestle and mortar containing 800 μL lysis buffer in a ZR BashingBead™ Lysis tube. The mixture was centrifuged at ≥12,000 x g for 1 min at 4 °C, and 400 μL of the supernatant was transferred to a Zymo-spin™ 111C column in a collection tube, and centrifuged for a further 8000 x g for 30 s. The RNA flow-through was washed with 320 μL of ethanol (95–100%), and centrifuged at ≥12,000 x g for 30 s in a Zymo-spin™ 11C collection tube. The RNA was re-suspended in 400 μL RNA prep buffer, centrifuged at 12,000 x g for 30 s, washed with RNA Wash buffer, and eluted with 30 μL of DNase/RNAses free water. A NanoDrop spectrophotometer was used to measure the RNA concentration at maximum absorbance of 260 nm, and the purity was assessed by measuring the 260/280 and 260/230 absorbance ratios. Using a Qubit 2.0 the concentration ranged from 100 ng/μL to 300 ng/μL and one microgram was run on a 1.5% agarose gel (70 V for 60 min). Total RNA was stored at − 80 °C.

### Library construction for next generation sequencing

Total RNA (1 μg per library) was used as the template to construct paired-end (PE) indexed Illumina libraries according to TruSeq RNA Library Preparation kit v2 (Illumina, San Diego, California) with modifications. To allow unbiased detection of polyadenylated and non-polyadenylated virus genomes [28, 29], oligo-dT purification was not performed. RNA fragmentation was done with Illumina fragment mix added to 19.5 μL of total RNA to make a volume of 70 μL. First strand cDNA was obtained using random hexamers and Superscript II reverse transcriptase. After double strand cDNA synthesis, ends were repaired by incubating in End Repair mix at 30 °C for 30 min. The End Repair mix contains 3′ to 5′ exonuclease to remove the 3′ overhangs while the polymerase activity filled in the 5′ overhangs. Thereafter, 3′ ends were adenylated and adaptors ligated to the 5′ (flow cell binding sequences) and 3′ end (barcode indexed adapters). The dsDNA was enriched by 15 PCR cycles at 98 °C for 30 s. Amplicon size and concentration of each library was verified using Qubit 2.0 and Bioanalyzer (RIN > 8) (Agilent 2000) (Agilent, Santa Clara, CA. USA). Barcoded libraries were normalized and pooled for multiplex sequencing. A pooled barcoded library (ten nanomolar) consisted of 24 biological samples, each at equal molar concentration. Libraries were sequenced in the Illumina MiSeq System using a 2 × 251 v2 kit including a 1% PhiX v 3 spike to generate paired-end reads (Illumina). Three flow cells were used, each for one pool of samples and 5 μl were loaded per lane. The sequencing was performed using Illumina MiSeq at the Biosciences Eastern and Central Africa–International Livestock Research Institute (BecA-ILRI) Hub in Nairobi, Kenya.

### RNA sequence processing and de novo assembly

Paired-end reads were de-multiplexed into individual samples using custom scripts at Biosciences Eastern and Central Africa-International Livestock Research Institute (BecA-ILRI) Hub, Nairobi, Kenya. Downstream bioinformatic analysis was done on high performance computing nodes at the Holland Computing Center (https://hcc.unl.edu) at the University of Nebraska-Lincoln. Sequence files were converted to fasta format, reads were evaluated using FastQC v0.11.2 [30], trimmed and filtered using Trimmomatic v0.36 [31] to remove adapter sequences, poly-N (≥10%) and low quality reads (Q ≤ 5). Simultaneously, Q30, GC-content and sequence duplication levels of the reads were calculated. For each individual sample, high-quality reads with a Phred score of 64, denoting high quality base calls were de novo assembled into contigs using Trinity v2.4.0 with Kmer size = 25 and other default parameters [32]. Contigs ≥ 200 bp were used for virus identification through BlastN (Additional file 1: Figure S1).

Alignment of the nearly complete genome contigs against their reference genomes was performed using Bowtie V2 under default parameters. Bam files were made for the resulting alignments. Samtools [33] and bcftools, with the criteria of MAPQ score > 10 and depth ≥ 3 for each read were used to generate a consensus sequence for each virus species or for a group of samples within a virus species. Visualizations were made using Integrative Genomic Viewer (v2.4.4) [34].

### Virus identification

BlastN was performed using de novo assembled contigs against a local Plant Virus Genome Database (PVGDB) containing 2166 plant virus genomes (http://www.ncbi.nlm.nih.gov/genome/viruses) (downloaded October 20, 2017) and the National Centre for Biotechnology Information (NCBI) “nr” databases. The cutoff was set at E-value ≤1 × 10^− 5^. The top accession, based on sequence similarity was obtained for each one of the contigs in our samples and used for virus identification. For each viral species, the most frequent accession was used as reference for alignment and to estimate sequence similarity. A virus was determined as present in a sample if at least one contig ≥ 200 bp with similarity ≥ 75% was detected (Additional file 1: Figure S1). Contigs matching viruses with lower similarity were not taken into consideration. Sequences not matching to any known virus or to the host were not analyzed further.

### Virus coverage maps

For each virus identified, a representative sample yielding a genome-length contig was chosen to determine read depth against reference genome sequences (MCMV, X14736.2; SCMV, JX188385.1; MSV, AF329878.1; and MYDV-RMV, MF974579.2). Reads were mapped onto each virus genome using Bowtie V2 [33]. The coverage indicates the percentage of the genome area covered by an average of three reads [35], while read depth refers to the number of reads covering the same sequence. Integrative Genomic Viewer (v2.4.4) was used for Graphical alignment visualization [34].

### Multiple sequence alignment and phylogenetic analyses

Sequences from NCBI (MCMV, SCMV, MSV and MYDV-RMV) used as reference were selected based on a combination of sequence identity (> 90%) and sequence coverage. For each virus individual contigs were aligned using multiple sequence alignment program for nucleotides and proteins (MAFFT, v7) using default parameters (http://mafft.cbrc.jp/alignment/server/phylogeny.html) [36]. Phylogenetic trees were generated as described [37]. Briefly, SplitsTree4 (http://www.splitstree.org) was used to generate splits networks, using the default settings. Distances were estimated by uncorrected P (match option for ambiguous bases) and network made by neighbour-net [38]. Further, to produce phylogenetic trees two runs of four Monte Carlo Markov Chain (MCMC) computations were run for 1,000,000 generations under a General-Time-Reversible (GTR) model with a gamma distribution of rate variation between sites Bayesian inference in MrBayes 3.2 [39]. Convergence and effective sample size were examined using Tracer to confirm that estimated sample sizes for each parameter exceeded 200, as recommended by the MrBayes manual. For each virus, the consensus trees and Bayesian posterior probability values at nodes were calculated with a 10% burn-in removed from each run.

### SCMV single nucleotide polymorphism

A single nucleotide polymorphism (SNP) analysis was done on the twenty samples with single contigs near complete SCMV genome. Illumina paired-end reads for each one of the samples were mapped against the SCMV reference genome (JX188385.1) using the BWA-mem option within BWA aligner (http://bio-bwa.sourceforge.net). To separate sequencing error from genomic variation, only reliable mapped reads were considered for SNP calling and unmapped reads were discarded. SNP positions within mapped reads were determined using samtools. VCFtools (http://vcftools.sourceforge.net) was used on the raw Variant Calling Format (VCF) files for the minimum depth (DP) 10 and SNP quality (Q) 30 to get high-quality SNPs. SNPs count was calculated using a 50 nt interval with the SNP density option within the VCFtools, and the plot generated in Excel.

## Results

### Identification of maize-infecting viruses

To gain insight on viruses associated with maize lethal necrosis and their genetic variation in Kenya, we conducted a metagenomics analysis based on next-generation RNA sequencing, *de-novo* assembly and identification of viruses in Kenya (Fig. 1b) through bioinformatics. Total RNA was used to construct paired-end reads from 68 individual samples representing sixteen counties. A total of 58.8 million reads were obtained, which were reduced to 57.2 million reads after trimming (Additional file 2: Table S1). After *de-novo* assembly of each individual sample, 1.95 million contigs were generated. After trimming, on average, each sample had 0.9 million reads that assembled in to 30,004 contigs with an average length of 340 bp (Additional file 2: Table S1). These contigs were used to determine the viruses present in using BlastN against the Plant Virus Genome Database and NCBI “nr” databases. Results clearly indicated the presence of four main viruses: *Maize chlorotic mottle virus* (MCMV)*, Sugarcane mosaic virus* (SCMV)*, Maize streak virus* (MSV) and *Maize Yellow Dwarf virus-RMV* (MYDV-RMV) (Fig. 1c). *Hubei Poty-like virus 1*, *Barley virus G*, *Scallion mosaic virus* and *Johnson grass mosaic virus* (JGMV) were detected in a smaller number of samples (Fig. 1d).

### Sequence depth and coverage of viruses identified

**Fig. 2:**
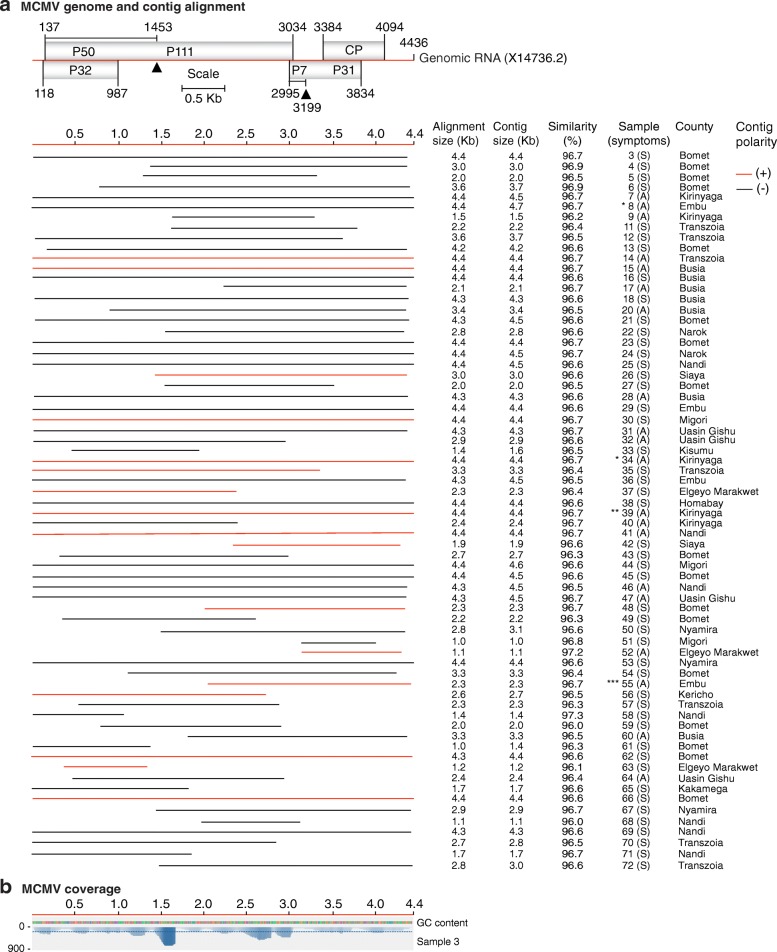
*Maize chlorotic mottle virus* (MCMV) genome organization and alignment of de novo-assembled contigs. Symptomatic (S) and asymptomatic (A) maize, cultivated (*) or wild (**) sorghum, or napier grass (***) were sampled. The county of origin is indicated after the sample number and symptoms. **a** MCMV genome organization. Coordinates are based on reference sequence number X14736.2. Open reading frames are represented by cylinders. Genomic RNA is represented by a solid line. Arrow heads mark the leaking termination codon in p50 and in p7. Red and black lines, to scale, represent contigs of positive or negative polarity, respectively, aligned to the reference. For every sample categorized as infected the longest contig is shown. Shorter, redundant contigs were not illustrated. Contig size, alignment size, and similarity (%) are indicated. **b** Genome coverage after reference based assembly using Bowtie v2 for one representative sample. Sequence depth is indicated on the left. GC content is color coded

**Fig. 3:**
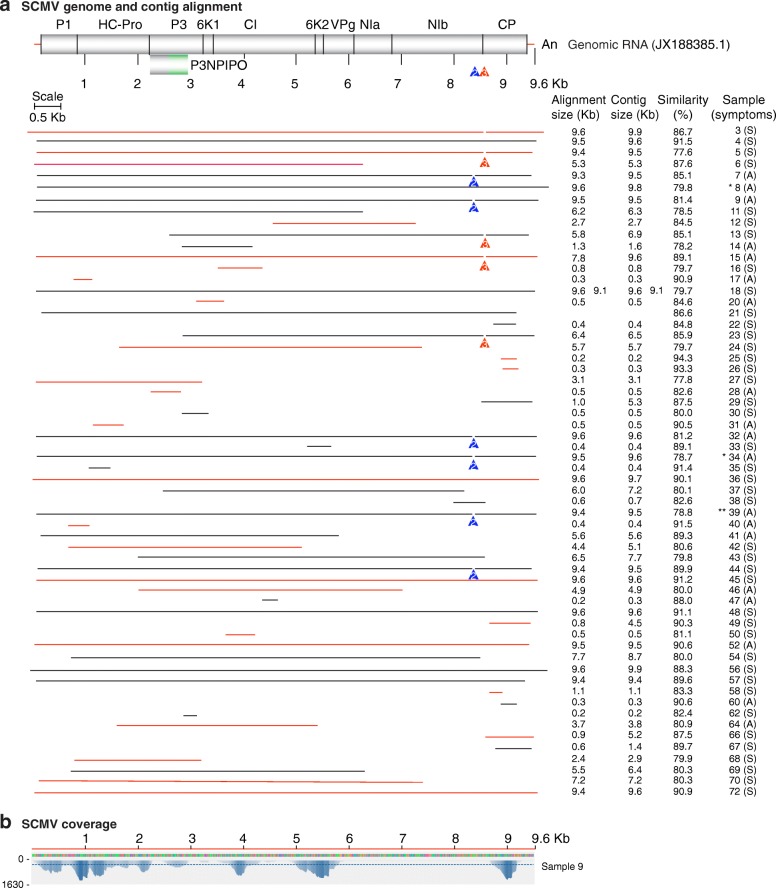
*Sugarcane mosaic virus* (SCMV) genome organization and mapping of de novo-assembled contigs. Labels are as in Fig. 2. **a** SCMV genome and polyprotein organization. Mature proteins are represented by cylinders. Coordinates are based on the Ohio isolate used as reference (JX188385.1). Every sample categorized as infected contributed one representative contig. A variable area was detected between nt 8500 and 8650. Colored arrowheads represent the location of two conserved deletions in the polyprotein coding sequence. A number 2 (group G2) indicates a 39 nt deletion (8487 to 8525) that resulted in an in-frame deletion of 13 amino acids at the C terminus of NIb. A number 3 (group G3) indicates a 45 nt deletion between nt 8487 to 8676 that resulted in a 15-amino acid deletion. In samples not marked (group G1), variation was observed without insertions or deletions. **b** Genome coverage after reference based assembly using Bowtie v2 for one representative sample

Our *de-novo* assembled single contigs were either short, similar or longer than the reference genomes (Additional file 3: Figure S2). Most of the gaps mapped to the 5’ and 3’ UTR. Contigs were selected for further analysis based on sequence length and alignment size (≥ 80% of the genome). Alignment size was calculated by subtracting the start from the end of the match using coordinates of the reference genome. In most cases, the alignment size was shorter than the contig size (Additional file 4: Table S2). The polarity of each contig was determined with respect to the reference genome. Graphical alignments for all samples and coverage maps were made for MCMV (Fig. 2), SCMV (Fig. 3), MSV (Fig. 5) and MYDV-RMV (Fig. 6). For one representative sample per virus, coverage and sequence depth at each nucleotide position were obtained. Sequence depth (reads per nt) for MCMV (Fig. 2b), SCMV (Fig. 3b), MSV (Fig. 5b) and MYDV-RMV (Fig. 6b) was at least 150, 200, 2000 and 150 reads, respectively.

Collectively, these results provide a clear identification with high similarity, coverage and depth for four main viruses: MCMV, SCMV, MSV and MYDV-RMV.

### Virus prevalence in Kenya and similarity to reference genomes

MCMV was the most prevalent virus in maize growing regions in Kenya. It was detected in all the 68 samples (Figs. 1b-c and 2a). *De-novo* assembled contigs ranged from 0.5 to 4.5 kb (Fig. 2a and Additional file 3: Figure S2) with > 96% similarity to the Kansas isolate (X14736.2) used as reference (Fig. 2a). Single contigs nearly covering the complete genome were obtained from 30 samples (Fig. 2a and Additional file 3: Figure S2). Respect to the reference genome, these contigs, lacked 2 to 297 nt at the 5’ end and/or 18 to 213 at the 3’ end.

**Fig. 4:**
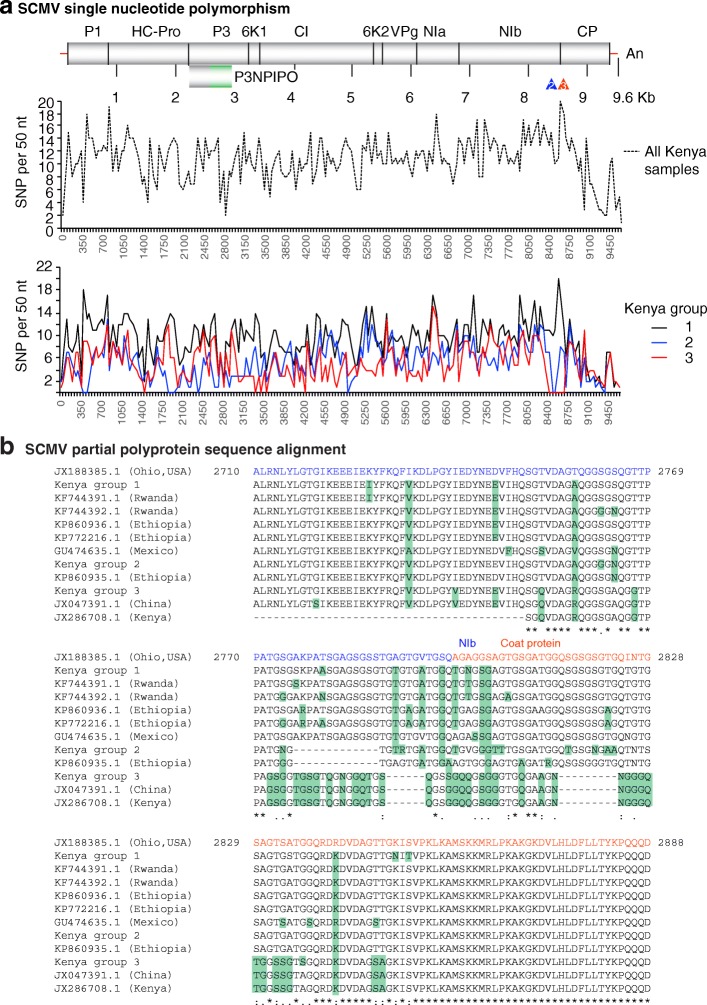
SCMV genetic variation. Coordinates are based on the Ohio isolate (JX188385.1). **a** SNP distribution across the SCMV genome for all samples and by genetic group. **b** Partial polyprotein sequence alignment, using MAFFT, of Kenya samples in variation groups 1, 2 and 3, and isolates from other parts of the world relative to the Ohio isolate. The coat protein detected in the original description of maize lethal necrosis in Kenya was used for comparison (JX286708.1) [6]. NIb and coat protein coding sequences are color coded blue and red, respectively. Green background indicates variation

SCMV was the second most prevalent virus in maize growing regions of Kenya (Fig. 1b-c). SCMV was present in 60/68 samples, contigs varied from 0.2 to 9.6 kb (Fig. 3a and Additional file 3: Figure S2) and had 77 to 95% similarity to the Ohio isolate (JX188385.1) used as (JX188385.1) (Fig. 3a). Single contigs close in size to the complete genome were obtained for twenty samples (Fig. 3a). These were 1 to 18 nt shorter at the 5’ end and/or 9 to 54 nt shorter at the 3’ end. Five contigs were longer than the reference genome (Fig. 3a and Additional file 3: Figure S2), and had 16 to 222 extra nt at the 5’ end and /or 1 to 129 extra nt at the 3’ end. A single nucleotide polymorphism analysis (SNP, see below) identified a variable area at the border between NIb and the coat protein (Fig. 4).

MSV was the third most abundant virus in maize growing regions of Kenya (Fig. 1b-c).

**Fig. 5:**
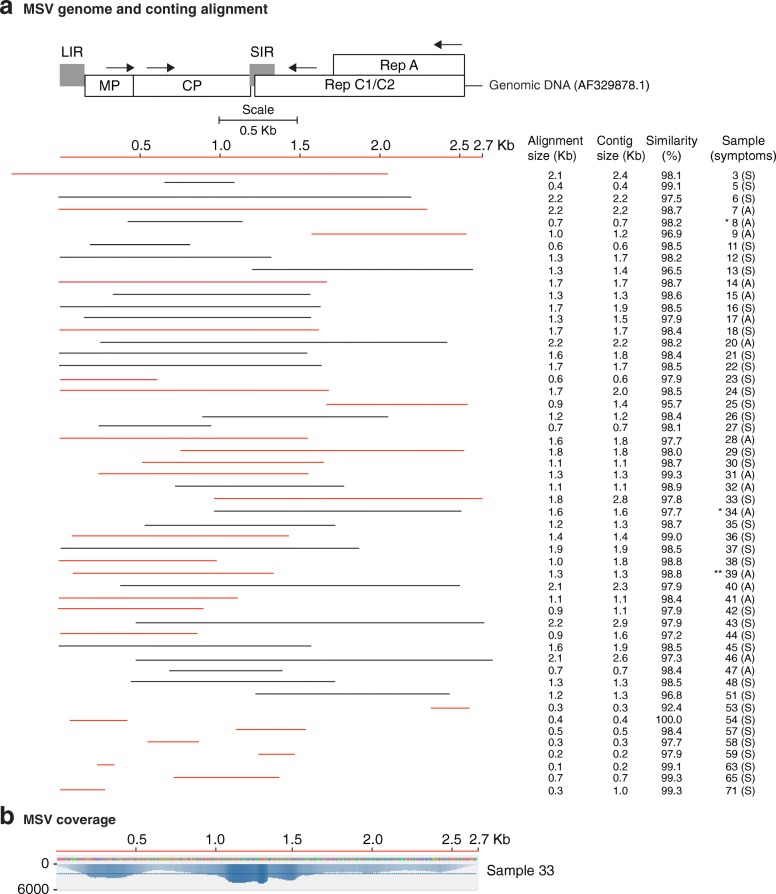
*Maize streak virus* (MSV) genome organization and alignment of de novo-assembled contigs. Labels are as in Fig. 2. **a** MSV genome organization. Open reading frames are represented by cylinders. Genomic DNA is represented by a solid line. Coordinates are based on reference sequence number AF329878.1. Large (LIR) and small (SIR) are represented by shaded boxes. Direction of transcription is indicated by arrows. Every sample categorized as infected contributed one representative contig. Shorter, redundant contigs were not illustrated. **b** Genome coverage after reference based assembly using Bowtie v2 for one representative sample

MSV was present in 52/68 samples, contigs varied from 0.2 to 2.6 kb (Fig. 5a and Additional file 3: Figure S2) and similarity to the reference genome (AF329878.1) was > 97% (Fig. 5a). Single contigs from eight individual samples were almost complete genomes. Two single contigs from two individual samples were longer than the reference genome (Additional file 3: Figure S2). Both had duplicated sequences at the 5’ end.

**Fig. 6:**
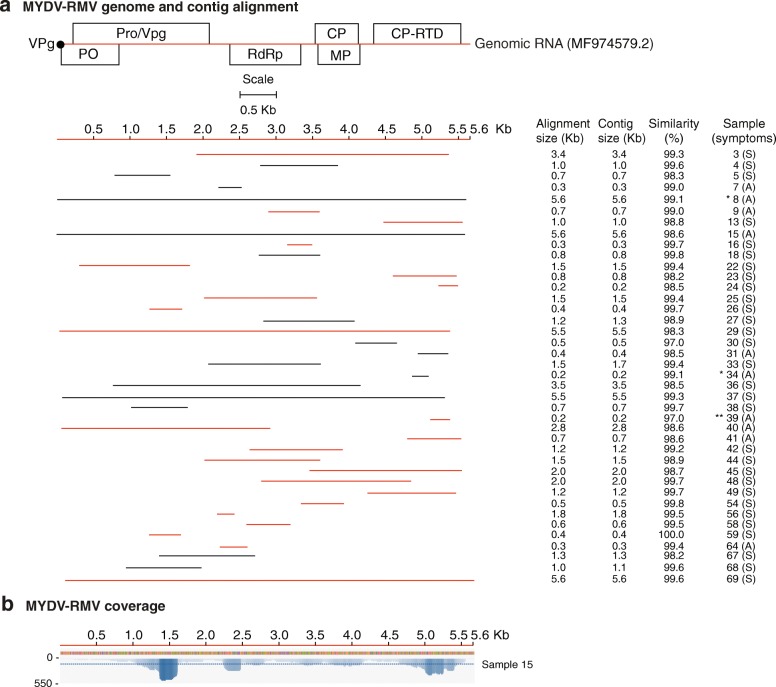
*Maize yellow dwarf virus* (MYDV-RMV) genome organization and alignment of de novo-assembled non-overlapping contigs from symptomatic (S) and asymptomatic (A) maize, cultivated (*) or wild (**) sorghum. Labels are as in Fig. 2. **a** MYDV-RMV genome organization and gene expression. Open reading frames are represented by cylinders. Genomic RNA is represented by a solid line. Coordinates are based on reference sequence number MF974579.2. Every sample categorized as infected contributed one representative contig. Shorter, redundant contigs were not illustrated. **b** Genome coverage after reference based assembly using Bowtie v2 for one representative sample

MYDV-RMV was the fourth most abundant virus in maize growing regions of Kenya (Fig. 1b-c). MYDV-RMV was present in 40/68 samples with contigs varying from 0.2 to 5.6 kb (Fig. 6a and Additional file 3: Figure S2) and > 96% similarity to the reference genome (MF974579.2) (Fig. 6a). Single contigs close to complete genome were obtained for five samples (Additional file 3: Figure S2). These contigs were 12 to 25 nt shorter at the 5’ end and/or 24 to 112 nt shorter than the reference genome at the 3’ end.

In addition to single contigs near genome length, for all four viruses described above, additional shorter overlapping contigs (Additional file 3: Figure S2) of opposite polarity were obtained (Additional file 4: Table S2) and used to generate genome length consensus sequences.

### Geographic distribution and profile of virus infections in maize

MCMV was detected in all the 68 samples (Figs. 1b and 2a) and including maize, sorghum and napier grass, and in all sixteen counties sampled. MCMV was detected in combination with one, two, three, or four other viruses in the 67 samples (Figs. 1b-c and 2a). Thirty of the 68 samples analyzed, included six samples from asymptomatic maize plants, and three sorghum samples, had a combination of four viruses: MCMV, SCMV, MSV and MYDV-RMV (Fig. 1c). In thirteen of the sixteen counties included in this study, at least one sample was detected containing all four viruses (Fig. 1b-c). In the three sorghum samples, MCMV was detected in combination with SCMV, MSV and MYDV-RMV (Fig. 1c). In the napier grass sample MCMV was detected alone. Interestingly, sorghum and napier grass plants showed no symptoms of virus infection at sampling (Fig. 1a).

The second most prevalent virus, SCMV, was found in 60 of the 68 samples. In all cases, SCMV was present in combination with MCMV, MSV and MYDV-RMV (Fig. 1c). MSV and MYDV-RMV were found in 52 and 40 samples, respectively. SCMV, MSV and MYDV-RMV were detected in all cases in combination with at least one other virus (Fig. 1c). SCMV and MSV were present in all sixteen counties, while MYDV-RMV was present in thirteen of the sixteen counties sampled.

These results show that MCMV, SCMV, MSV and MYDV-RMV are widely distributed across maize growing counties in Kenya (Fig. 1b).

### Other viruses infecting maize

Four potyviruses and one polerovirus were detected in a smaller number of samples (Fig. 1d). *Hubei Poty-like virus 1* (19 samples), *Scallion mosaic virus* (7 samples), *JGMV* (5 samples), and Iranian *JGMV* (2 samples) are potyviruses. *Barley virus G (11 samples)* is a polerovirus.

The *Hubei Poty-like virus 1* reference genome (NC_032912.1) is 9356 nt long. The longest contig we obtained was 9323 nt long and was 77.3% similar to the reference (sample 48). The highest similarity (87.3%) to the reference genome was obtained for a 206-bp contig (sample 68). The *Scallion mosaic virus* reference genome (NC_003399.1) is 9324 nt long. The longest contig we obtained was 962 nt long and was 80.0% similar to the reference (sample 20). The highest similarity (89.3%) to reference genome was obtained for a 271-bp contig (sample 68). The JGMV reference genome (NC_003606.1) is 9779 nt long. The longest contig we obtained was 1535 nt long and was 75.0% similar to the reference (sample 46). The highest similarity (85.6%) to reference genome was obtained for a 967-bp contig (sample 30).

Collectively, these results show that *Hubei Poty-like virus 1*, *Scallion mosaic virus*,

*JGMV*, Iranian *JGMV*, and *Barley virus G* are part of the virus complex infecting maize in Kenya and their genetic composition is distant from isolates described before (Fig. 1d).

### Low genetic diversity of maize chlorotic mottle virus in Kenya

**Fig. 7:**
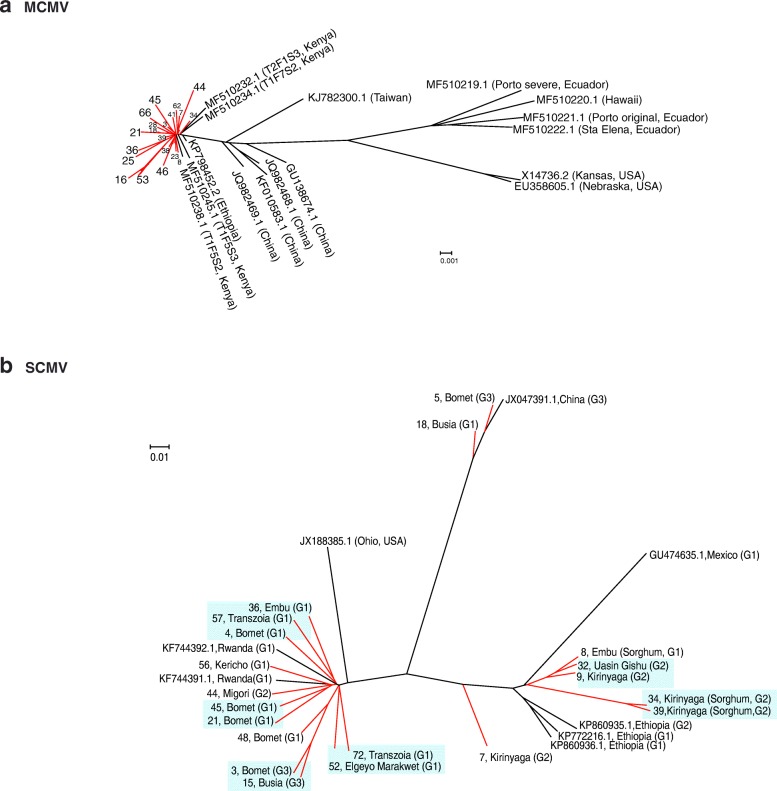
Phylogeny of MCMV (**a**) and SCMV (**b**). Phylogenetic trees were generated using Bayesian inference in Mr. Bayes 3.2. Scale bar represents nucleotide substitution per site. For SCMV, G1, G2 and G3 correspond to genetic variation and groups described in Fig. 4. Kenya samples described in this study are colored in red and identified by a number and the county of origin. Unless indicated otherwise, samples came from maize. Green background indicates clusters formed by Kenya samples

Thirty contigs from this study (Additional file 5) were used for a phylogenetic analysis that included 16 sequences from GenBank representing MCMV world wide variation [37]. MCMV sequences from Kenya were at least 96% similar to the Kansas isolate (X14736.2) used as reference (Fig. 2a). In agreement with world wide variation [37], our results showed a clear distribution of MCMV isolates in different clades based on their geographic origin (Fig. 7a). Kenya samples described here clustered in the clade containing isolates from East Africa, close to isolates from China and away from isolates from the American continent (Fig. 7a). Within our Kenya samples, there was no correlation with the county or host of origin. One sample (number 16) lacking 15 nt and 205 nt at the 5’ end and 3’ end, respectively, showed the most distance from the African cluster (Fig. 7a). Results described here and before [37] show that there is low genetic variation in the MCMV population in Kenya.

### Genetic variation in *Sugarcane mosaic virus*

Twenty SCMV sequences from this study (Additional file 6) and eight complete genomes from GenBank representing different parts of the world were used on a phylogenetic analysis. Previously described isolates from Rwanda, Ohio, China, Mexico, and Ethiopia formed clearly separate clusters (Fig. 7b). Samples described here were distributed in six different clusters containing at least two members. Six other samples were placed individually near clusters formed by Kenya samples or isolates from other parts of the world. Consistent with variation in similarity (77 to 94%) to the Ohio isolate (JX188385.1) (Fig. 3a), this result, suggests that there is genetic variation in the SCMV population in Kenya.

Alignment to the Ohio isolate showed that out of the twenty near genome length contigs, eleven had a gap that mapped to the border between NIb and the coat protein (Fig. 3a). To understand this variation, a single nucleotide polymorphism analysis (SNP) was carried out using the twenty SCMV contigs near genome length used in the phylogenetic analysis (Additional file 6). SNP were estimated at a 50 nt interval. Although there was additional variation across the genome, the most variation mapped to nt 8500 to 8650 which corresponds to the border between NIb and the coat protein (Fig. 4a) and include the gaps observed in the alignment to the Ohio isolate (Fig. 3a). Nucleotide (Additional file 7: Figure S3) and amino acid sequence alignment of the NIb and coat protein separated our Kenya samples into three distinct groups (Fig. 4b). Group one (samples 4, 8, 18, 21, 36, 45, 48, 52, 56, 57 and 72) was the most frequent, and no nucleotide insertions or deletions were observed (Additional file 7: Figure S3). However, there were nucleotide and amino acid substitutions at the C terminus of NIb and at the N terminus of the coat protein (Fig. 4b). Group two (samples 7, 9, 32, 34, 39 and 44) had a 39-nt deletion (8487 to 8525) (Additional file 7: Figure S3) that resulted in an in-frame deletion of 13-amino acids at the C terminus of NIb (Fig. 4b). Group three (samples 3, 5, and 15) had low similarity and a 45-nt deletion between nt 8487 to 8676 (respect to the reference) (Additional file 7: Figure S3) that resulted in a 15-amino acid deletion. An SNP analysis for samples within each group clearly distinguished the three groups described above and showed that most of the variation maps to nt 8500 to 8650. At that interval, groups 2 and 3 harbor a deletion. However, additional variation occurs across the rest of the genome (Fig. 4a, lower panel). Interestingly, in this analysis, the least variation was observed at the PIPO coding sequence (Fig. 4a, middle panel). PIPO is a highly conserved protein in potyviruses with an essential role in virus movement [40].

To further characterize genetic variation in SCMV from Kenya, the polyprotein was obtained for the consensus sequence of each group and aligned to the polyprotein for isolates representing several parts of the world. No complete genome has been described for SCMV from Kenya to date. The coat protein sequenced in the original description of maize lethal necrosis in Kenya was used for comparison (JX286708.1) [6]. Consistent with the SNP and nucleotide sequence alignment, variation in the SCMV polyprotein formed three groups. Respect to the Ohio isolate, group one has several amino acid substitutions at the C terminus of NIb and at the N terminus of the coat protein (Fig. 4b). Similar variation was observed for two isolates from Rwanda, two from Ethiopia and one from Mexico. In addition to amino acid substitutions similar to those in group 1, group two has a deletion of 13-amino acids at the C terminus of NIb. The same deletion is present in one isolate from Ethiopia (Fig. 4b). In addition to amino acid substitutions similar to those in group 1, group three has a 15-amino acid deletion. Six amino acids mapped to the C terminus of NIb and nine mapped to the N terminus of the coat protein (Fig. 4b). This deletion is present in one isolate from China and in the isolate from the original description of maize lethal necrosis in Kenya (JX286708.1) [6] (Fig. 4b).

In the phylogenetic analysis, samples that cluster together belong to the same group based on variation between NIb and the coat protein (Fig. 7b). However, some samples from the same group were placed away from the cluster (Fig. 7b), suggesting that there is additional variation along the SCMV genome. In support of this observation, the SNP analyses identified other sources of variation in the SCMV genome (Fig. 4a, lower panel).

Samples from the counties of Kirinyaga and Uasin Gishu clustered near isolates from Ethiopia, while samples from Bomet, Migori, Transzonia and Kericho clustered near isolates from Rwanda (Fig. 7b). Thus, there is correlation between geographic location and genetic diversity of SCMV populations in Kenya. However, samples from Busia, and from Embu, were in separate clusters.

Variation described above for SCMV in Kenya is unlikely to be sequencing error, because similar deletions are present in published SCMV isolates and because variation mapped to a common area in all samples analyzed (Fig. 3a). Furthermore, geographic distribution of genetic variation was not random. Of the six samples in group two, four came from the county of Kirinyaga: two from maize (samples 7, 9) and two from sorghum (samples 34 and 39) (Fig. 3a). Of the three samples in group three (samples 3, 5, and 15), two (3 and 5) came from maize samples from the county of Bomet and one from the county of Busia.

Results described above show that SCMV from Kenya exhibits high genetic variation that formed six clusters based on genome sequence. Kenya samples and isolates from other parts of the world can be divided into at least three groups based on nucleotide and amino acid sequence at the C terminus of NIb and N terminus of the coat protein (Fig. 4b).

### Maize streak virus exhibits low genetic variation

**Fig. 8:**
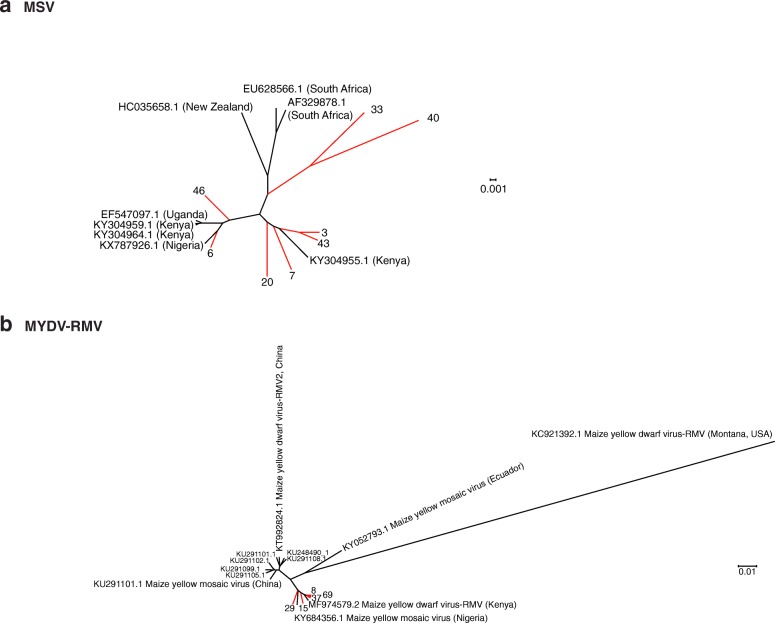
Phylogeny of MSV (**a**) and MYDV-RMV (**b**). Phylogenetic trees were generated using Bayesian inference in Mr. Bayes 3.2. Scale bar represents nucleotide substitution per site. Kenya samples described in this study are colored in red and identified by a number

MSV described in this study showed 96 to 100% similarity to the South African isolate (AF329878.1) used as reference (Fig. 5a). Eight contigs representing almost complete genomes (Additional file 8) and eight from GenBank were used for a phylogenetic analysis. Six of our Kenya contigs clustered near isolates from Uganda, Nigeria, and previously described Kenya isolates (Fig. 8a). Two samples (33 and 44) from Kenya clustered separately near isolates from New Zealand and South African isolates. These and previous results [41] show low genetic variation in the MSV population in Kenya.

### Polerovirus complex infecting maize

Based on five contigs (Additional file 9) from this study and seventeen sequences from GenBank, a phylogenetic tree was obtained for MYDV-RMV. *Maize yellow mosaic virus* and *Maize yellow dwarf virus*-RMV2 were included for comparison. Sequences from Kenya obtained in this study were 97 to 100% similar to (Fig. 6a) and four clustered near the MYDV-RMV reference (MF974579.2), while two clustered near *Maize yellow mosaic virus* (MaYMV) isolate from Nigeria (Fig. 8b). However, the similarity between MYDV-RMV and MaYMV is 98.67%.

These results and the widespread distribution of MaYMV in Rwanda [21] suggest that in Kenya there is a complex of closely related poleroviruses that include *Maize yellow dwarf virus*-RMV and *Maize yellow mosaic virus*, and possibly others, such *Barley virus G* which was detected in 11 of the 68 samples (Fig. 1d).

## Discussion

Maize lethal necrosis disease is caused by the synergistic co-infection of MCMV and a member of the *Potyviridae*. Synergism has been confirmed for SCMV [6, 12], WSMV [18], and JGMV [19]. Recently, the polerovirus *Maize yellow mosaic virus* (MaYMV) was detected in maize plants showing lethal necrosis-like symptoms in Rwanda [21]. In the analysis described here, the polerovirus *Maize yellow dwarf virus* (MYDV-RMV) was found to be widely distributed in Kenya (Figs. 1b and 6), and the polerovirus *Barley virus G* was detected in 11 of the 68 samples analyzed (Fig. 1d). MYDV-RMV was always found as part of a complex that included MCMV and SCMV, or MCMV, SCMV and MSV. The wide distribution of poleroviruses infecting maize in Rwanda [21] and in Kenya (Fig. 1b) suggests the possibility of a synergistic interaction between MCMV and a polerovirus to cause maize lethal necrosis, and may contribute to the variation on virus-induced symptoms observed in the field (Fig. 1a).

The molecular mechanisms of viral synergism in maize lethal necrosis remain to be determined. One model is that maize lethal necrosis is mediated by silencing suppressors encoded by the co-infecting viruses. In support of this model, the synergistic interaction between potyviruses and *Potato virus X* (PVX) and *Cucumber mosaic virus* (CMV) is mediated by silencing suppression activity of potyviral HC-Pro [42, 43]. Consistent with this model, SCMV and WSMV encode RNA silencing suppressors HC-Pro and P1, respectively [44, 45]. Several, poleroviruses, including MaYMV encode PO, a strong RNA silencing suppressor [46]. These observations are consistent with a role for maize-infecting poleroviruses in maize lethal necrosis.

However, no silencing suppressor has been described for MCMV [47] or MSV (a Mastrevirus) [48]. Interestingly, in *Wheat dwarf virus* (a Mastrevirus) replication-associated proteins are silencing suppressors [49], which suggest that MSV harbors silencing suppressor proteins. Further investigation is needed to determine the role of silencing suppression, and the contribution of poleroviruses and MSV to maize lethal necrosis.

There is ambiguity with respect to the scientific name given to poleroviruses infecting maize. In 2013, the first species was named *Maize yellow dwarf virus* (MYDV-RMV) [50]. Two different isolates from China were named *Maize yellow mosaic virus* (MaYMV) [46] and *Maize yellow dwarf virus*-RMV2 (MYDV-RMV2) [51], while an isolate infecting sugarcane in Nigeria was named *Maize yellow mosaic virus* (MaYMV) [52], and an isolate infecting maize in Kenya was renamed as *Maize yellow dwarf virus*-RMV (MF974579.2). Three near genome length polerovirus contigs from maize and one from sorghum described here were most closely related (99% similarity) to *Maize yellow dwarf virus*-RMV (Figs. 6b and 8b). However, one near genome length contig from maize was most closely related to *Maize yellow mosaic virus* (MaYMV) (Fig. 8b), the most prevalent virus infecting maize in Rwanda [21]. These observations suggest that a complex of closely related poleroviruses infect both maize, sorghum, and possibly other species in East Africa.

In East Africa, ELISA [19, 21, 22] and RT-PCR [9] procedures have provided inconsistent detection of SCMV. Sequencing analysis described here and before [6] show that the SCMV present in Kenya (Figs. 3 and 4) and in Rwanda [9] is distantly related to isolates from other parts of the world (Fig. 7b). Interestingly, our results showed that most of the variation occurs between the C terminus of NIb and the N terminus of the coat protein (Figs. 3a and 4). Both nucleotide and amino acid variation was observed in all twenty Kenya samples that provided near complete genome contigs (Fig. 4 and Additional file 7: Figure S3). Based on this variation, Kenya samples, and isolates from other parts of the world, were divided into three groups. Nucleotide substitutions that resulted in several amino acid substitutions in both NIb and the coat protein was the most frequent event (group 1, 11 samples) (Figs. 3a and 4b). However, in the other nine samples, in-frame deletions resulted in a 13-amino acid deletion at the C terminus of NIb (group 2, 6 samples) (Fig. 4b), or in a 15-amino acid deletion distributed between the C terminus of NIb and the N terminus of the coat protein (group 3, 3 samples) (Fig. 4b).

In members of the Potyviridae, NIb is required for virus replication, while the coat protein participates in virion assembly, cell-to-cell and systemic movement [53]. The effect of amino acid substitutions and deletions at the C terminus of NIb and at the N terminus of the coat protein on virus pathogenicity remain to be determined. Presence of these deletions in SCMV isolates from other parts of the world suggest that viruses harboring these deletions are pathogenic. Consistent with this hypothesis, in *Wheat streak virus* (Family Potyviridae, genus Tritimovirus), a genetic analysis using an infectious clone showed that deletions at the N terminus of the coat protein are tolerated and mutants cause more severe symptoms than the wild type virus in several hosts [54, 55]. Alternatively, in the absence of co-infecting viruses, in SCMV deletions between the C terminus of NIb and the N terminus of the coat protein may be lethal.

Polyprotein alignment showed that the 15-amino acid deletion observed in three Kenya samples (group 3) is present in one isolate from China and in the isolate reported in the original description of maize lethal necrosis in Kenya (JX286708.1) [6] (Fig. 4b). Amino acid variation at the C terminus of NIb and at the N terminus of the coat protein in Kenya group 1 is similar to variation in two isolates from Rwanda [9], two from Ethiopia and one from Mexico. Additionally, the 13-amino acid deletion observed in 6 samples from Kenya (group 2) is present in a SCMV isolate from Ethiopia (Fig. 4b). Furthermore, three complete genomes have been described from Ethiopia [11]. In our analysis, they formed a clear cluster between the China and Mexico isolate (Fig. 7b). Interestingly, one isolate from Ethiopia harbors the 13-amino acid deletion described here for 6 Kenya samples (Fig. 4b, group 2). Cloning, sequencing, and restriction digestion analysis of SCMV infecting sugarcane in India [56] and maize in Brazil [57] showed that the N terminus of the coat protein is hypervariable. A similar analysis showed genetic diversity in SCMV coat protein sequence in Cameroon and Congo [58]. These observations show that SCMV harbors a hypervariable region between NIb and the coat protein.

Variation at the C terminus of NIb and N terminus of the coat protein in SCMV could explain inconsistent detection of SCMV by ELISA [19, 21, 22], and failure to detect SCMV in Rwanda [9] (similar to group 1) by RT-PCR using primers designed for Kenya group 3. These observations highlight the need to raise antibodies against African isolates and universal primers to detect SCMV. Alternatively, or in addition, plants showing maize lethal necrosis symptoms could be infected by other potyviruses. In addition to SCMV, other potyviruses found in Kenya samples include were *Hubei Poty-like virus 1*, *Scallion mosaic virus* and *JGMV* (Fig. 1d). Interestingly, JGMV in combination with MCMV, causes maize lethal necrosis [19]. The role of other potyviruses in maize lethal necrosis remains to be determined.

Screening of germplasm and commercial hybrids for resistance to maize lethal necrosis has focused on MCMV and SCMV [5, 24–26]. The widespread distribution of MYDV-RMV, MSV, and possibly JGMV (Fig. 2b) [21] highlights the need to include other viruses in breeding programs seeking to develop virus-resistant cultivars or hybrids for East Africa.

Multiple sources of virus may contribute to maize lethal necrosis epidemic. Soil and seed transmission is possible for both MCMV and SCMV [5, 59]. Additionally, both potyviruses and poleroviruses are transmitted by aphids [50, 60]. MCMV is transmitted by several species of beetles in the family *Chysomelidae* [61] and by western flower thrips (*Frankliniella occidentallis*) [62]. Despite lacking visible viral symptoms, the three sorghum samples we analyzed (Fig. 1c) contained MCMV, SCMV, MSV and MYDV-RMV. Similarly, one asymptomatic napier grass sample contained MCMV (Fig. 2). Consistent with these observations, several grass species and sorghum cultivars were determined to be asymptomatic hosts for MCMV, SCMV and WSMV [5, 59]. Thus, sorghum, napier grass and possible other grass species are virus reservoirs for insect vectors to spread the viruses to maize. Several factors, including genotype, plant age and days after infection at the time samples were collected, may contribute to the absence of symptoms in our sorghum and napier grass samples. Further experimentation is needed to determine the response of sorghum and napier grass to viruses that cause maize lethal necrosis and to understand their role as alternate hosts.

Although MCMV, SCMV, WSMV and JGMV are present, maize production is not reduced due to maize lethal necrosis in the United States [63, 64]. After the initial detection in Kansas and Nebraska in the 1970’s [65], maize lethal necrosis was managed by a combination of agronomic practices that included crop rotation, removal of alternate hosts, and use of hybrids tolerant to MCMV or SCMV [65, 66]. Epidemiological models and field surveys show that growing maize continually results in an increase of virus inoculum [5, 63]. Consistent with these observations, crop rotation could reduce the prevalence and delay infection [63]. However, in East Africa, maize is grown year-round during two growing seasons, underscoring the need to develop integrated management strategies to slow the spread and damage caused by maize lethal necrosis. The strategy must include identification and deployment of virus tolerant germplasm, seed sanitation and distribution programs, identification and removal of alternates, and insect vector control, and the establishment of a systematic surveillance program. SCMV in Kenya are genetically different to isolates from other parts of the world (Fig. 4). Thus, phytosanitary regulations could be implemented on maize and sorghum grain imports. These measures require rapid and reliably diagnosis. Sequences described here provide a solid foundation to develop global, directed multiplex nucleic acid-based methods to diagnose MCMV, SCMV, MSV, MYDV-RMV and closely related viruses.

## Conclusions

The maize lethal necrosis epidemic in Kenya is complex. In addition to MCMV and SCMV, several other potyviruses and possibly poleroviruses are involved (Fig. 1). Sorghum, napier grass and possibly other plant species participate as alternate hosts. SCMV is widely distributed in Kenya (Fig. 1b) and consists of numerous strains that are genetically different to isolates from other parts of the world (Fig. 7b). SCMV harbors a hypervariable region at the border between NIb and the coat protein. These observations provide a solid foundation to design integrated disease management strategies, and have potential to impact breeding programs aiming to developing SCMV resistance, diagnostic protocols, and quarantine regulations.

## Additional files

Additional file 1: **Figure S1.** A schematic representation of sampling strategy, RNA sequencing, analysis, and virus identification. (PDF 301 kb)

Additional file 2: **Table S1.** Description of samples used for identification of viruses associated to maize lethal necrosis in Kenya by a metagenomic analysis. (XLSX 1273 kb)

Additional file 3: **Figure S2.** Size and frequency of de novo-assembled representative contigs with high similarity to known viruses. In total 68 samples were sequenced and analyzed. Contig size is represented in 0.5 Kb increments in the X axis. The Y axis represents the number of contigs per size class. For each virus, the number of samples categorized as infected is indicated. **a** MCMV. **b** SCMV. **c** MSV. **d** MYDV-RMV. (PDF 278 kb)

Additional file 4: **Table S2.** Virus identification by BlastN against the Plant Virus Genome Database and contig alignment. (XLSX 1123 kb)

Additional file 5: MCMV contigs used for phylogenetic analysis. (FASTA 132 kb)

Additional file 6: SCMV contigs used for phylogenetic analysis. (FASTA 193 kb)

Additional file 7: **Figure S3.** Partial nucleotide sequence alignment of Kenya samples in group 1, 2 and 3 relative to the Ohio isolate of SCMV (JX188385.1). The coat protein detected in the original description of maize lethal necrosis in Kenya was used for comparison (JX286708.1) [6]. Alignment was generate using MAFFT. NIb and coat protein coding sequences are color coded blue and red, respectively. Coordinates are based on the Ohio isolate (JX188385.1). In group 1 no nucleotide insertions or deletions were observed. Nucleotide substitutions resulted in amino acid substitutions. Group two, had a 39 nt deletion (8487 to 8525) that resulted in an in-frame deletion of 13 amino acids. Group 3, had low similarity and a 45 nt deletion between nt 8487 to 8676 that resulted in a 15-amino acid deletion. (PDF 256 kb)

Additional file 8: MSV contigs used for phylogenetic analysis. (FASTA 20 kb)

Additional file 9: MYDV-RMV contigs used for phylogenetic analysis. (FASTA 27 kb)
