# Supplementary figures and images for: Metagenomic analysis of viruses associated with maize lethal necrosis in Kenya
Source: Virol J. 2018 May 23;15:90. doi: 10.1186/s12985-018-0999-2 (PMC5966901; doi:10.1186/s12985-018-0999-2)

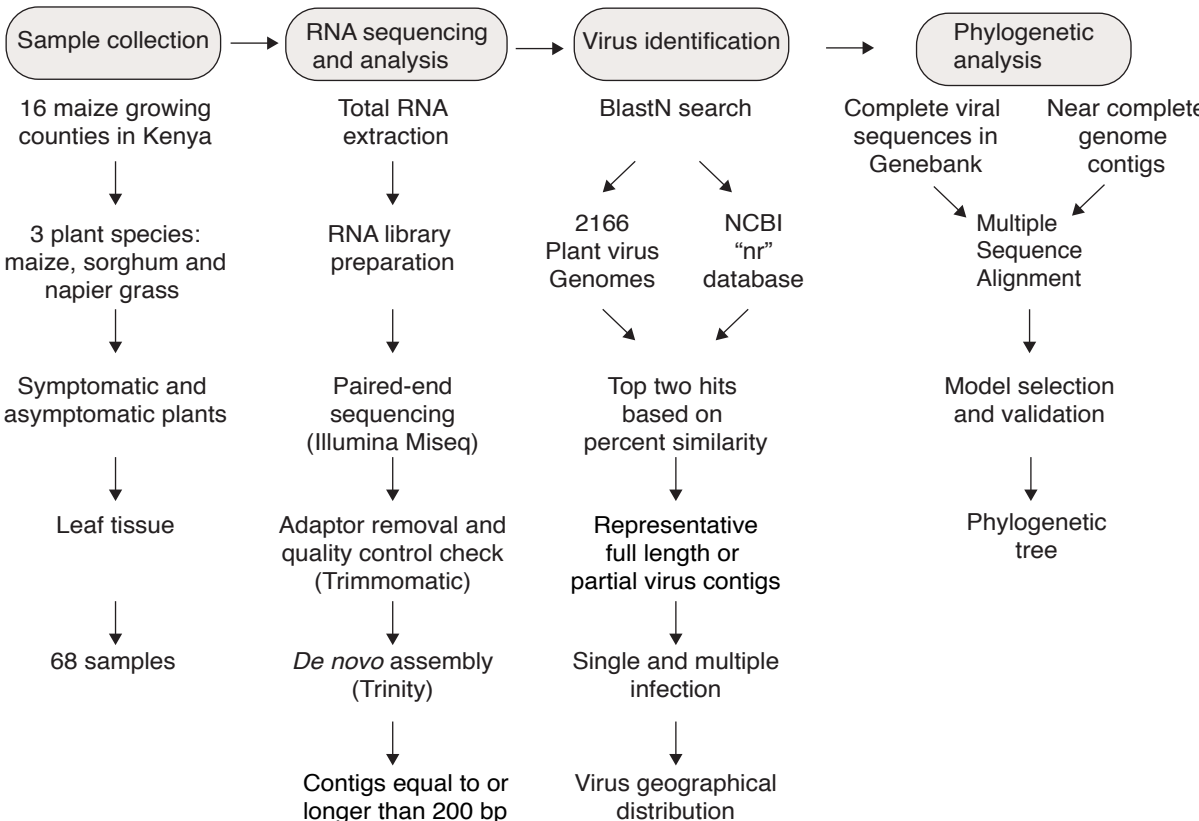

Supplement: Supplementary file 1 — Figure S1. A schematic representation of sampling strategy, RNA sequencing, analysis, and virus identification. (PDF 301 kb) [file 12985_2018_999_MOESM1_ESM.pdf]

**a MCMV**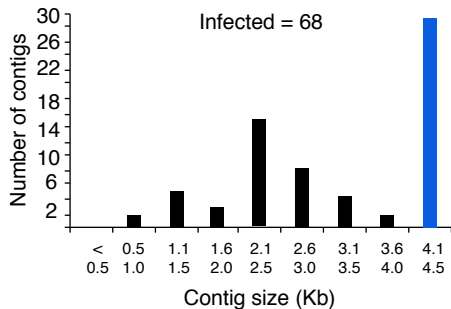**b SCMV**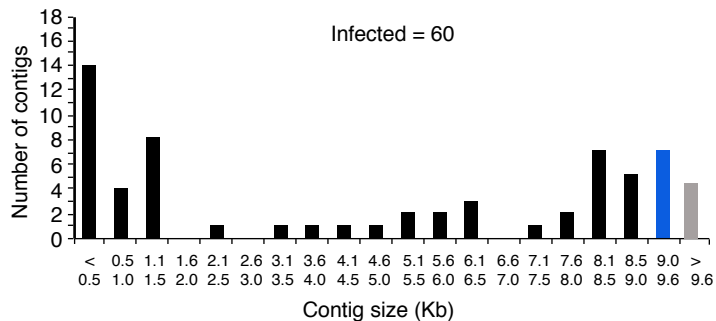**c MSV**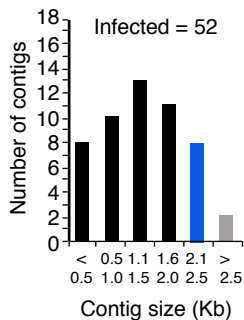**d MYDV-RMV**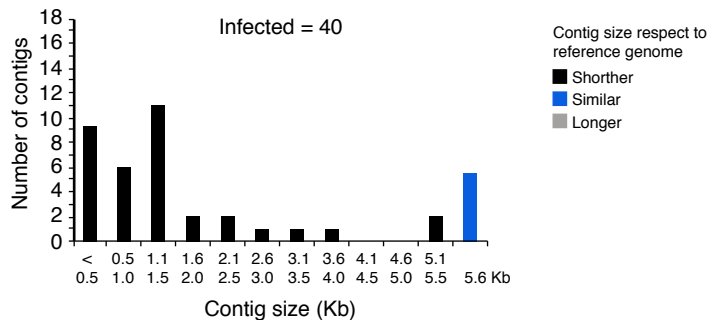

Supplement: Supplementary file 3 — Figure S2. Size and frequency of de novo-assembled representative contigs with high similarity to known viruses. In total 68 samples were sequenced and analyzed. Contig size is represented in 0.5 Kb increments in the X axis. The Y axis represents the number of contigs per size class. For each virus, the number of samples categorized as infected is indicated. a MCMV. b SCMV. c MSV. d MYDV-RMV. (PDF 278 kb) [file 12985_2018_999_MOESM3_ESM.pdf]
