# Supplementary material for: Metagenomic analysis of viruses associated with maize lethal necrosis in Kenya
Source: Virol J. 2018 May 23;15:90. doi: 10.1186/s12985-018-0999-2 (PMC5966901; doi:10.1186/s12985-018-0999-2)
Supplement: Supplementary file 7 — Figure S3. Partial nucleotide sequence alignment of Kenya samples in group 1, 2 and 3 relative to the Ohio isolate of SCMV (JX188385.1). The coat protein detected in the original description of maize lethal necrosis in Kenya was used for comparison (JX286708.1) [6]. Alignment was generate using MAFFT. NIb and coat protein coding sequences are color coded blue and red, respectively. Coordinates are based on the Ohio isolate (JX188385.1). In group 1 no nucleotide insertions or deletions were observed. Nucleotide substitutions resulted in amino acid substitutions. Group two, had a 39 nt deletion (8487 to 8525) that resulted in an in-frame deletion of 13 amino acids. Group 3, had low similarity and a 45 nt deletion between nt 8487 to 8676 that resulted in a 15-amino acid deletion. (PDF 256 kb) [file 12985_2018_999_MOESM7_ESM.pdf]

```
Ohio            8397    CAGTCGGGAACTGTTGATGCAGGTACACAAGGAGGCAGTGGAAGCCAAGGAACAACACCA 8456
Kenya group 1           CAATCGGGAACAGTTGATGCAGGTGCACAAGGCGGCAGCGGAAGCCAAGGAACAACACCA
Kenya group 2           CAATCGGGAACAGTTGATGCAGGCGCACAAGGAGGCGGCGGAAATCAAGGAACAACACCG
Kenya group 3           CAATCTGGTCAAGTTGACGCAGGGAGACAGGGCGGTAGCGGTGCTCAAGGAGGCACGCCA
JX286708.1 (Kenya)      ---TCTGGTCAAGTTGACGCAGGGAGACAGGGCGGTAGCGGCGCTCAAGGAGGCACACCG
                        ** **    ***** *****   *** ** **  * **    ******   ** **

Ohio            8457    CCAGCAACAGGCAGTGGAGCAAAACCAGCCACCTCAGGGGCAGGATCTGGTAGTAGCACA 8516
Kenya group 1           CCAGCAACAGGTAGCGGATCGAAACCAGCGGCTTCAGGAGCAGGATCTGGTAGCGGAACA
Kenya group 2           CCAGCAACAGGTAACGGAACAGG-------------------------------------
Kenya group 3           CCAGCAGGAAGTGGAGGCACTGGATCTGGCACTCAAGGCAATGGGGGTCAGA--------
JX286708.1 (Kenya)      CCAGCAGGAAGTGGAGGCACTGGATCTGGCACTCAAGGCAATGGGGGTCAGA--------
                        ******  * *    **  *

                                         NIb           Coat protein
Ohio            8517    GGAGCTGGAACTGGTGTAACTGGAAGTCAAGCAGGGGCTGGCGGTAGCGCTGGACGGGA 8576
Kenya group 1           GGGACTGGAACCGGTGCAACTGGAGGCCAAACAGGAAATGGTAGTGGTGCTGGAACAGGA
Kenya group 2           --AACCAGAACTGGTGCAACTGGAGGCCAAACAGGAGTTGGTGGAACTACAACAGGA
Kenya group 3           ----CGGGATCCCAAGGAAGTAGTGGTCAAC------------------------AAGGG
JX286708.1 (Kenya)      ----CGGGATCCCAAGGAAGTGGCGGTCAAC------------------------AAGGG
                            *  ** *    * ** * *  * ***                           **

Ohio            8577    TCCGGAGCAACCGGAGGCCAAYCAGGATCTGGAAGTGGCACTGGACAGATTAACACGGGT 8636
Kenya group 1           TCTGGAGCGACCGGAGGCCAATCAGGATCTGGAAGTGGCACTGGACAGACTGGCACAGGC
Kenya group 2           TCTGGAGCGACCGGAGGTCAGACAGGATCTGGAAATGGTGCTGCACAGACCAACACGAGC
Kenya group 3           TCCGGTGGGGGCACTGGTCAAGGAGCAGCTGGAAACAA---------CGGCGGAGGTCAG
JX286708.1  (Kenya)     TCCGGTGGGGGCACTGGTCAAGGAGCAGCTGGAAACAA---------CGGCGGAGGTCAG
                        ** ** *    *   ** **   ** * ******

Ohio            8637    TCAGCAGGAACTAGTGCAACAGGAGGCCAAAGAGATAGGGATGTGGATGCAGGTACAACA 8696
Kenya group 1           TCAGCAGGAACTGGTTCAACGGGAGGCCAGAGAGATAAGGATGTGGATGCAGGTACAACA
Kenya group 2           TCAGCAGGAACTGGTGCAACGGGAGGCCAGAGAGATAAGGATGTAGATGCAGGTACAACA
Kenya group 3           ACAGGAGGCTCTAGTGGGACATCTGGTCAGAGAGATAAGGACGTTGACGCAGGCTCGGCT
JX286708.1 (Kenya)      ACAGGAGGCTCTAGTGGGACAGCTGGTCAGAGAGATAAGGACGTTGACGCAGGCTCGGCT
                        *** ***  ** **    **    ** ** ******* *** ** ** *****  *  *
```
